# An evaluation of an Australia-based home Burglary prevention program

**DOI:** 10.1057/s41284-022-00355-0

**Published:** 2022-09-16

**Authors:** Matthew Manning, Gabriel T. W. Wong, Melody Ip

**Affiliations:** ANU Centre for Social Research and Methods, The Australian National University, Canberra, ACT 2601 Australia

**Keywords:** Burglary, Household security, Repeat burglary, Household security assessment, Burglary prevention devices, Sense of security

## Abstract

In response to an increase in the number of burglaries in the Australian Capital Territory (ACT) from 2014 to 2020, the ACT government funded the development of a home-based Burglary prevention program. The aim of the program is to improve household security particularly for those properties at heightened risk of victimisation and re-victimisation. The program consisted of security assessments of properties and, based on assessments, installation of security devices for eligible program clients. Results from the evaluation reveal that the program produces positive benefits overall in terms of enhanced security, reduced risk of re-victimisation, improvement in perceived sense of personal security, and positive economic return on investment.

## Background

In recent years (2015–2019), Australia overall has seen the number of burglary victimisations remain relatively stable with an annual average of 178,276 victims of burglary crimes (defined as unlawful entry with intent) (Australian Bureau of Statistics 2021).^1^ However, Canberra (Australian Capital Territory (ACT)), Australia’s capital city, saw an increase in the number of burglaries from 2014 to 2020. As well as the obvious economic costs associated with residential burglary, victims of burglary can experience serious psychological impacts including distress, insecurity and fear of repeat victimisation (Macguire 1980). Such fear of a repeat is real as research suggests that the risk of burglary victimisation is disproportionally borne by former victims of burglary (Zimring and Hawkins 1995). Budd (2001) found that a burglary record was one of the best predictors of repeated burglary victimisation – 20 percent of burglary victims were burgled at least once in the same year, while seven percent of those who experienced victimisation were burgled more than twice in the same year. Farrell and Pease (2013) observed that rates of repeated burglaries were higher than the repeat rates of other crimes against households and individuals such as damage to property and assault. In short, victims of burglary bear a relatively high chance of being re-victimised compared to other crime victims. Robinson (1998) indicates that burglary victims are most likely to be re-victimised soon after their first victimisation (Lister et al. 2004).

The literature on burglary prevention tends to focus on several crime prevention approaches that have the potential to reduce the risk of victimisation and repeat victimisation. The evaluation of the interventions which were reviewed by Grove et al. (2012) mainly focused on a combination of: (i) social prevention methods such as neighborhood watch-based schemes (Bennett et al. 2008) and burglary prevention initiatives such as the dissemination of crime prevention advice to potential victims (Lister et al. 2004); as well as (ii) crime prevention through environmental design (CPTED) which emphasises the adoption of security devices to harden targets such as installing locks, bolts and gates to protect property (Jeffery 1971).

The increased use of the above-mentioned approaches has seen a marked reduction in the volume of crimes in most countries over time (Tilley et al. 2011). For example, with respect to social prevention, Stokes and Clare (2019) reveal that victimisation information can be utilised to reduce the opportunity for burglary by means of working in a targeted and timely manner as well as circulating burglary crime prevention information in order to alter residents’ behaviour. Apropos CPTED, Tilley et al. (2011) observed a reduction in domestic burglary by 58% between 1995/1996 and 2008/2009. Focusing on repeat victimisation, Grove et al. (2012) conducted a meta-analysis of 31 interventions in which 22 studies were aimed at preventing repeat residential burglary victimisation. Repeat victimisation was found to have declined in 81% of studies. On average, repeat victimisation was reduced on average by approximately 60% among studies where changes in repeat victimisation could be observed. Other important findings drawn from this review include: (i) carefully tailored situational crime prevention measures appear to have the greatest effect with regards to reducing crime; (ii) effective implementation is a key factor in intervention success; and (iii) advice and education for victims of crime are often not effective.

While target hardening has been employed internationally and widely cited as an effective strategy for burglary reduction (Weisel 2002; Hirschfield 2004; Reynald 2014; Tilley et al. 1999), several initiatives specific to government subsidised target-hardening of homes include: Kirkholt burglary reduction project (Pease 1991; Tilley 1993); Liverpool Citysafe (Newton et al. 2008); the Safer Cities programme (Ekblom et al. 1996); the Secured by Design (SBD) initiative (Armitage 2000; Cozens et al. 2004); the Burglary Reduction Initiative as part of the government’s Crime Reduction Programme (Maguire 2004; Tilley 2004); Locks for Pensioners (Mawby and Jones 2006); and the Distraction Burglary initiative (Thornton et al. 2003). Many of the above-named projects, many of which have been located in the UK, including the Kirkholt project, prioritized recent victims of burglary. This was in recognition that reducing repeat victimisation was the most effective way of reducing overall burglary rates (Farrell and Pease 1993).

In spite of the above evidence, Grove et al. (2012) highlight the need for additional research into the prevention of repeat victimisation of different crimes including burglary. Extending our knowledgebase on victimisation and repeat victimisation prevention in different jurisdictions, including those where few evaluations have been conducted, will allow for differences to be captured with regard to contextual variation (e.g. demographic differences, policing models, implementation strategies). Further, additional studies will supplement the current literature base to assist in identifying the potential moderators that will assist criminal justice organisations to better target those factors that are most likely to reduce the risk of victimisation.

### Theory and approaches to reduce burglary

The main references of our study include the CPTED approach, broken window theory, routine activity theory and the rational choice perspective. CPTED is a multidisciplinary methodology consisting of target hardening, natural surveillance, territoriality, defensible space, formal organized surveillance, access control and activity program support (Crowe 1991). For example, a housing development with limited natural surveillance enhances the risk of residential burglary by reducing the offender’s probability of being detected (Cozens et al. 2018; Monchuk et al. 2019). The average number of storeys in a building, street density and proportion of residential area within a district or suburb also influences the burglary rate (Sohn 2016). Burglary prevention interventions that adopt CPTED principles create an impression of a neighborhood in good maintenance and thus discourage residential crime. For instance, by removing graffiti as quickly as possible (Lee et al. 2016). The impact of modifying the environment in order to reduce burglary victimisation is also supported by perception studies (Armitage 2018; Armitage and Monchuk 2017). For instance, Armitage (2018) showed different images of residential housing to incarcerated prolific burglars and found that the design of residential buildings influences their decision to burgle or not.

The broken windows theory articulates that an unrepaired broken window signals a lack of ownership and creates an environment in which further crime and disorder may be encouraged (Kelling and Wilson 1982). The image of dilapidated housing could influence the perception of disorder and may serve as a signal to potential offenders that there is little guardianship and hence the probability of being caught is low (Cozens et al. 2001; Shaw and Gifford 1994; Zhang and McCord 2014). Jang et al. (2008), for example, reveal that broken windows enforcement (i.e. enforcement upon minor offenses and “uncivil” behaviors) positively influenced the clearance rates of burglaries.

Routine activity theory (RAT), developed by Cohen and Felson (1979), identifies the mechanism by which crime occurs as a result of the convergence of a suitable target, a potential offender, and lack of a capable guardian. RAT has been useful for crime prevention practitioners in understanding the nature of property crime and identifying precautions and measures that reduce crime opportunities (Argun and Dağlar 2016). According to Tseloni et al. (2004), direct indicators of guardianship consistently predict the mean number of burglaries where occupied households have a lower risk of burglary victimisation and households left empty on a regular basis increase the risk of burglary. The influence of routine activities on patterns of burglary can be observed during the outbreak of COVID-19. Here, containment policies implemented in response to the outbreak led to a swift transformation in people’s routine activities, which shifted burglaries away from residential areas to non-residential locations (Felson et al. 2020).

The rational choice perspective proposes that a rational actor makes choices that are influenced, in part, by anticipating the costs and benefits associated with the alternatives of either committing or not committing crime (Cornish and Clarke 1986). For example, burglars may weigh up the anticipated costs and benefits of undertaking burglary based on the perceived risk of detection, the probability of conviction, and the perceived severity of punishment (Manning 2018). In addition, the burglar’s logic will also incorporate the difficulty or effort required in undertaking the crime. For example, the offender may target a property with relatively lower security over a property with better security measures (Snook et al. 2011). Effort-related attributes influence burglars’ decisions to target suitable households. Specifically, households closer to where a burglar resides are more likely to be chosen given the shorter distance to travel and the unique knowledge possessed by the burglar of that area (e.g. escape routes, places for hiding and intelligence for evading detection) (Vandeviver et al. 2015). Further, residences which are less accessible and pose difficulties for easy escape are significantly less likely to be selected as a target for burglars given the high risks involved (Langton and Steenbeek 2017).

### The current study

**Fig. 1:**
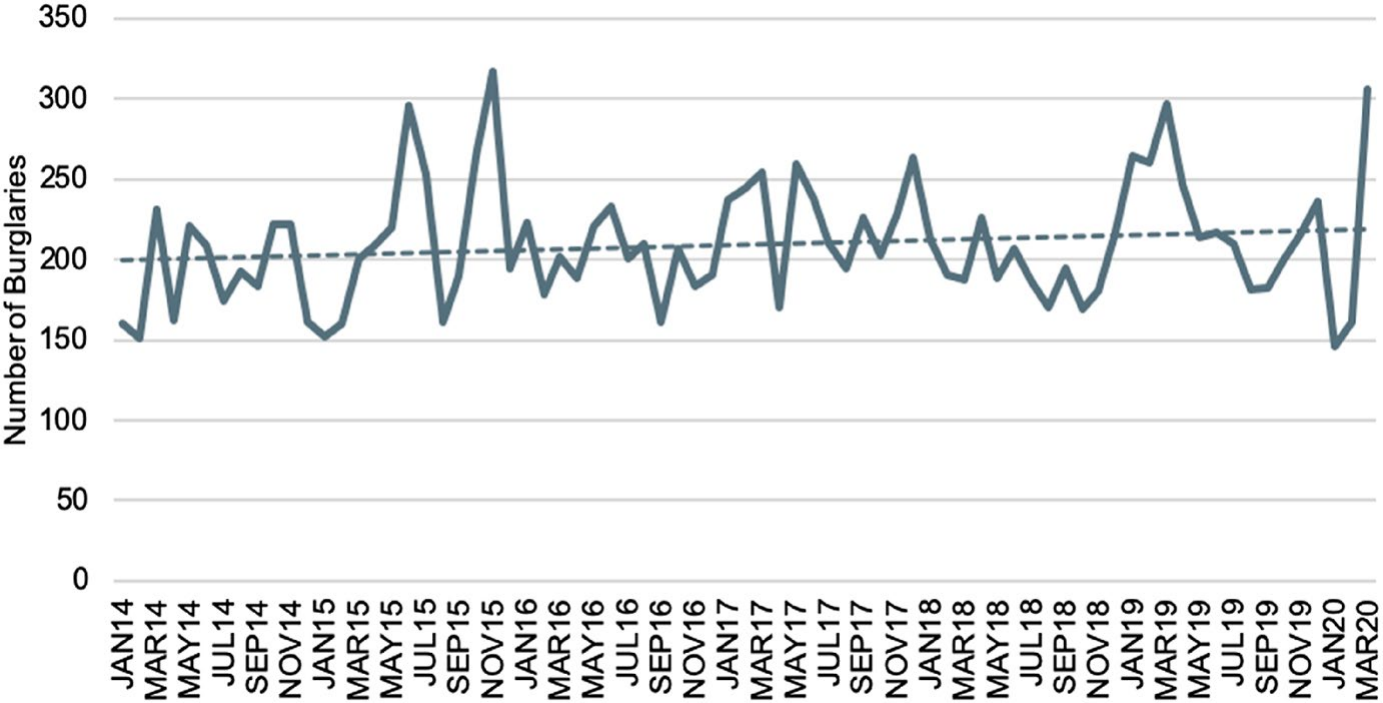
Number of burglaries in the ACT (2014–2020). *Source* Australian Federal Police. (2020). Crime statistics and data. Retrieved from https://www.policenews.act.gov.au/crime-statistics-and-data/crime-statistics

In response to an increase in the number of burglaries that occurred in the ACT (see Fig. 1), and an understanding of the financial and psychological impact that is felt by victims and also the knowledge that probability of victimisation is increased among those identified as at risk (e.g. those previously victimised) (Kleemans 2001), the ACT Government enacted the SafeHome Program.

The SafeHome program aims to improve household security for properties in the ACT that are vulnerable or at a heightened risk of property crime victimisation or re-victimisation. Program entry eligibility was based on recent victimisation where victims were typically referred to the program by ACT Policing. To assist in reducing the probability of revictimization, security experts provide home safety assessments highlighting areas where security can be increased mainly through target hardening (e.g. installations of window locks and gates). The program logic was based on the CPTED model. Minor modifications were made to properties where the residents’ annual household income was below AUD40,000. Households with an income above that threshold were provided with the expert assessment but were not eligible for the government funding of the modifications proposed by the expert. There was limited funding to the SafeHome program and, hence, the program could only afford making minor modifications in eligible households. Practical workshops are also delivered to community groups, presenting ideas and strategies to improve household security. An important component of this program is an evaluation of its impact on: (1a) the security of people’s homes; (1b) the likelihood of them becoming a victim/repeat victim of property crime; (1c) a client’s perception of safety and psychological conditions^2^; and (2) the economic benefit or loss that may be associated with the program. This paper provides results obtained from service user surveys, official re-victimisation statistics from ACT Police, SafeHome pre- and post-service assessment on physical security, and SafeHome bi-annual performance reports.

## Method

### Outcome 1a: The impact of SafeHome on the security of clients’ homes

To measure the impact of SafeHome on household security, a survey was administered by the service provider (SupportLink) to elicit responses regarding the change, if any, in overall household security as a result of the program. Survey data were collected during September to December 2020.^3^ The service provider reached out to 65 respondents, with 56 (86%) agreeing to participate. Three of the 56 participants were excluded due to substantial non-responses (over 90%), resulting in 53 valid participants.

Participants were asked what combination of household security measures were in place prior to their involvement in the program and what security measures were subsequently adopted. Specifically, the presence of any burglary security devices in the respondent’s home were measured (e.g. door or lock shields; window locks; security signage; security cameras). Information on changes to the environment (e.g. removal of obscuring foliage) and defensible space (e.g. clear boundaries demonstrating ownership) was also sought.

These data allow for the identification of improvements in household security as a result of the program, where households were disaggregated into four categories of security level: (i) no security—no use of any of the above mentioned security measures, environmental changes or use of defensible space; (ii) less than basic—households with one or more security measures, but not having both window and door double locks or deadbolts in place; (iii) basic—window locks and door double locks or deadbolts; and (iv) enhanced—basic plus at least one other device, environmental changes and use of defensible space. The evidence on the effectiveness of anti-burglary security devices suggests that the more enhanced the home security the less likely for a household being a victim of property crime (Manning and Fleming 2017).

### Outcome 1b: the impact of SafeHome on the likelihood of experiencing repeat victimisation

The ideal way to measure the likelihood of experiencing repeat victimisation would be to employ a quasi-experimental design using ACT police crime data at the household level during the reference period (2017/2018 to 2019/2020). With the use of local crime data, information on whether households in the population (i.e. SafeHome Program participants and eligible comparison group households) experienced repeat victimisation within a given time period could be ascertained. Information on the frequency of repeat victimisation could also be identified. To achieve this, recent crime data on those participating households and eligible households which did not participate would be required. A logistic regression could then be conducted to predict the likelihood of repeat victimisation and measure the impact of the program on this outcome. This method would also allow for the estimation of the number of avoided repeat victimisations. Here, the difference/change in probability of repeat victimisation between intervention and comparison groups (i.e. the cumulative incidence in the exposed group minus that of the unexposed group) would be applied to the sample as an average net effect of the program.

In this study, a comparison could only be made (due to availability of data) between the aggregated repeat burglary rate among program participants and the average rate of repeat burglary in the ACT, which serves as a baseline. The repeat victimisation rate in the participant group was elicited via the service user survey data (incorporated into Outcome 1a). Victimisation data, from 2017/18 to 2019/20, regarding the ACT repeat burglary rate at the most aggregated level was obtained from the ACT Police (i.e. number of re-victimised households divided by the number of initially victimised households within a specified time period—within 12 months).^4^

Here, a difference in re-victimisation rate may be attributed to the impact of the program. If re-victimisation was reduced, we would expect to have a lower re-victimisation rate among program participants when compared to the ACT average. The number of reduced victimisations was multiplied by the societal savings of an avoided burglary (using converted UK Home Office cost data (Heeks et al. 2018)) to represent the financial benefits of avoided repeat burglary for each participating household.

### Outcome 1c: the impact of SafeHome on clients’ perception of safety and psychological conditions

Validated survey questions from existing literature were adopted to measure participants’ perception of safety. Questions were included in the same survey as 1a and 1b to capture any changes in perceived safety/benefits of additional security measures. Specifically, questions were posed to capture participants’ sense of safety, in terms of personal safety/asset security, before and after participating in the program and modifying home security measures (e.g. installation of locks/alarms/gates). Answers to the questions were required to fit into one of the categories: ‘very safe’, ‘safe’, ‘neither safe nor unsafe’, ‘unsafe’, ‘very unsafe’, and ‘not applicable’. These questions concern service users’ sense of security while walking in the neighbourhood and at home during the day and at night.

Participants’ affliction with negative emotions and psychological conditions were captured using retrospective self-report survey responses. Specifically, participants were asked how often they experienced negative feelings (i.e. stress, sleeplessness, depression, anxiety and unhappiness) as a result of the state of household security before SafeHome modifications were made. They were also asked to attribute improvements in emotions and psychological conditions post-SafeHome modifications.

### Outcome 2: cost–benefit analysis of SafeHome

Project administrative budget data (July 2018–June 2020) from ACT Justice and Community Safety Directorate and the service provider were utilised to calculate the numerator in a cost–benefit ratio. This includes information about the costs under the categories of staffing, administration, travel, workshops, and home security assessment modifications.

Monetised benefits (denominator in cost–benefit ratio) were estimated using: (i) savings of avoided burglary; and (ii) non-market benefits on perceived improvement in security and wellbeing using the contingent valuation method via willingness-to-pay (WTP). The derived costs and benefits were entered into the Smart Cost–Benefit Tool (Manning and Wong 2019) to provide economic evidence on the impact of the program.

We examined participant’s WTP for all additional security devices implemented and adopted as a result of the program. These devices might have been provided/installed by the SafeHome service provider or by the client. In the majority of cases, the ACT Government paid for the purchase and installation of these devices via the service provider. To examine people’s WTP for the installation of security devices in their home, we needed to elicit a monetised response on their WTP for a device in order to reduce the probability of their house being burgled. A participant could theoretically place a high value, no value or any value in between on a particular device based on their perceived usefulness of that device in protecting their person and property.

As stated above, we employed a stated preference WTP measure, specifically contingent valuation. Contingent valuation is a well-established survey technique that is utilised to derive monetary estimates of the economic return on investment (Weatherly et al. 2014). This approach is commonly used in studies of WTP (see Cohen et al. 2004; Kling et al. 2012) and involves asking participants what they would be willing to pay for a particular good or service. Respondents were required to provide a WTP response to specific security devices, the face-to-face security assessment and overall modifications to reduce the risk of burglary re-victimisation.

The other measure used here is adapted from Rowe and Wood (2013), who asked organizations how much they would be willing to pay to improve the effectiveness of their security by X%. We modified the percentage improvement to either 50% or 90%. Specifically, we asked “Overall, how much would you be willing to pay for the services provided by the SafeHome if it were to reduce your risk of being a target of future burglary that you may experience by 50%/90%?” This allowed us to assess whether a percentage improvement played a role in participants’ WTP estimates.

## Results

### Demographics of survey participants

There were 53 valid survey responses. Table 1 provides information on participant age, gender, income, educational attainment, employment, marital status and type of residence. The majority of respondents were aged 55 and above and were predominantly female. In addition, the majority of participants resided, at the time of interview, in a house.

**Table 1:** Participant demographics

	Count^+^	%
Age		
18–25	0	0.00
26–34	8	15.09
35–44	10	18.87
45–54	1	1.89
55–64	8	15.09
65–74	11	20.75
75+	14	26.42
Prefer not to answer	1	1.89
Gender		
Male	6	11.32
Female	47	88.68
Annual household income		
Under $40k	21	39.62
$40–$100k	13	24.53
Over $100k	3	5.66
Prefer not to answer	16	30.19
Educational attainment		
Primary	1	1.89
Some secondary	14	26.42
Secondary (Year 12)	9	16.98
Tertiary/college	21	39.62
Prefer not to answer/unknown	8	15.09
Employment		
Employed full-time	7	13.21
Employed part-time	10	18.87
Not in the labour force (e.g. retired, performing home duties, attending an educational institution, permanently unable to work, etc)	31	58.49
Prefer not to answer	5	9.43
Marital status		
De facto	3	5.66
Divorced	5	9.43
Married	8	15.09
Never married	1	1.89
Separated	7	13.21
Widowed	9	16.98
Single	11	20.75
Prefer not to answer	9	16.98
Type of residence		
House	39	75
Semi-detached house*	4	7.69
Townhouse	7	13.46
Flat or apartment	2	3.85

*House that shares one common wall with the next house

^+^The total may not always be 53 due to missing data

#### Outcome 1a results: the impact of SafeHome on the security of clients’ homes

The level of household security includes four categories: (i) no security; (ii) less than basic; (iii) basic; and (iv) enhanced. We expected that all participants, to some degree, would be motivated to improve their household security to avoid initial, or further victimisation. A substantial improvement of security can be observed via a shift in respondent’s security configuration from ‘no security’ or ‘less than basic’ security before SafeHome to ‘basic’ or ‘enhanced’ security after SafeHome (Table 2). The most common additional modifications observed in this study to improve security level from ‘basic’ to ‘enhanced’ include the installation of security cameras and burglary alarms.

**Table 2:** Level of security before and after SafeHome

Level of security	Before count (%)	After count (%)
No security	7 (13.5)	0 (0)
Less than basic	23 (44.2)	1 (1.9)
Basic	22 (42.3)	34 (65.4)
Enhanced	0 (0)	17 (32.7)

Data reveal that the involvement of SafeHome is critical for household security improvement as the majority of respondents reported that they would not have been able to implement the recommended changes without their assistance. As seen in Table 3, the main reason for respondents not being able to implement changes to their security is based on their financial position.

**Table 3:** Whether or not respondents would be able to make security changes or improvement by themselves

	Count^a^	%
Yes	14	29.8
No	33	70.2
Due to financial restrictions	19	40.4
Due to physical restrictions/limitations	8	17.0
Due to the lack of knowledge/consideration	4	8.5
Due to property restriction	2	4.3

^a^The total may not always be 53 due to missing data

Focusing on modifications carried out by SafeHome, the most common changes involve the installation or repair of security screens, the installation or repair of a deadbolt, deadlatch or patio bolt, and the installation of door viewers. Changes and modifications were typically made when there was an absence of the security measure during the initial assessment. Those measures that were already in place during the initial assessment typically did not require installation or repair (see Table 4). It appears that modifications were made to address security issues that could plausibly reduce the risk of re-victimisation but also be achieved within the program budget.

**Table 4:** Presence, absence and modification of security measures

	Presence of the security measure during the initial assessment Count (%)	Absence of the security measure during the initial assessment Count (%)	N/A Count (%)	Modification carried out by SafeHome^a^ Count
Bushes, shrubs and trees are trimmed and maintained allowing visibility of doors and windows	45 (86.5)	6 (11.5)	1 (1.9)	0
House number is easy to see from the street both day and night	48 (92.3)	3 (5.8)	1 (1.9)	1
Sufficient lighting preferably sensor lighting covering front of house and potential hiding places	37 (71.2)	14 (26.9)	1 (1.9)	0
Spare keys are not left outside your home	43 (82.7)	8 (15.4)	1 (1.9)	6
Letter box is secured and mail is cleared daily	39 (75)	12 (23.1)	1 (1.9)	7
Automatic timers used for lighting and radios, etc	47 (90.4)	3 (5.8)	2 (3.8)	0
Solid core doors are in place on all external doors	49 (94.2)	3 (5.8)	0 (0)	2
Doors are fitted with a deadbolt/deadlatch or patiobolt	19 (36.5)	31 (59.6)	2 (3.8)	29
Security screens are fitted and in good working order	13 (25)	38 (73.1)	1 (1.9)	33
Effective door viewer is installed	19 (36.5)	29 (55.8)	4 (7.7)	24
Locks/clamps are on all windows	37 (71.2)	14 (26.9)	1 (1.9)	12
Valuables are concealed and not visible from windows	51 (98.1)	0 (0)	1 (1.9)	0
Neighbourhood Watch /security warning/beware of dog signs/stickers are attached to windows/gates	27 (51.9)	22 (42.3)	3 (5.8)	7
Back and side gates are secured	30 (57.7)	15 (28.8)	7 (13.5)	10
Tools, ladders or other garden items are stored in a secure place	43 (82.7)	4 (7.7)	5 (9.6)	0
The garden shed and garage is well secured and windows are covered	38 (73.1)	6 (11.5)	8 (15.4)	2
Meter box is secured against interference	22 (42.3)	22 (42.3)	8 (15.4)	4
Property are engraved or marked and a full inventory of the serial numbers are maintained	23 (44.2)	26 (53.8)	1 (1.9)	2

The total may not always be 53 due to missing data

^a^Modification can be made to improve existing security measure

#### Outcome 1b results: the impact of SafeHome on the likelihood of experiencing repeat victimisation

Survey respondents reside within 38 suburbs of Canberra. According to official crime statistics 1807 households within the 38 suburbs were burgled during the reference period and 56 households were re-victimised within 12 months, reflecting a re-victimisation rate of approximately 3.1%. According to survey responses, one out of 53 households within the 38 suburbs was re-victimised, reflecting a re-victimisation rate of approximately 1.9%. While the suburb-specific re-victimisation rate on attempted burglary was not available, one of the survey respondents reported a burglary attempt which was believed to be unsuccessful due to the enhanced security measures implemented as the result of SafeHome.

#### Outcome 1c results: the impact of SafeHome on clients’ perception of safety and psychological conditions

##### Sense of security

Results show that SafeHome participants acquired an improved sense of security. Specifically, a significant improvement in self-reported feeling of safety was found for participants walking around their neighbourhood or staying at home during the day and at night. Before SafeHome, approximately half of the participants felt unsafe walking in their neighbourhood (53%) and staying at home (49%) (Table 5). After SafeHome evaluation and modifications, a large number of participants no longer felt unsafe walking in their neighbourhood (65%) or staying at home (87%). The SafeHome program was shown to have a small to medium effect on improving participants’ overall sense of security when walking in their neighbourhood (*d* = 0.419)^5^ and a very strong effect for enhancing the feeling of safety for participants staying at home (*d* = 0.926). Finally, 68% of participants were at least somewhat concerned about potential break-ins and re-victimisation before SafeHome. This number declined to 35% after SafeHome.

**Table 5:** Participants’ sense of security

	Before SafeHome assessment and modifications		After SafeHome assessment and modifications	
	Mean (*M*)	Standard deviation (SD)	*M*	SD
Walking in the neighbourhood during the day	− 0.085	1.299	0.391	1.085
Walking in the neighbourhood at night	− 0.581	1.239	− 0.186	1.220
Overall walking in the neighbourhood	**− 0.767**	**2.359**	**0.163**	**2.092**
Staying at home during the day	− 0.154	1.274	0.962	1.073
Staying at home at night	− 0.412	1.252	0.792	1.116
Overall staying at home	**− 0.569**	**2.468**	**1.755**	**2.018**

Bolding relates to overall figures to assist in their easy identification

##### Other perceived impacts of victimisation on participant’s life

According to survey responses, approximately 70% of all valid responses (not including the ‘do not know’ response) of participants attributed their affliction with stress, sleeplessness, depression, anxiety and unhappiness to their former state of household security (Table 6). Twenty-two participants (out of 51 valid responses) also reported that these feelings hindered them from doing or engaging in activities they wanted to do in life.

**Table 6:** Whether participant’s former state of household security contributed to given negative feelings/conditions, Count (%)

	Stress	Sleeplessness	Depression	Anxiety	Unhappiness
Never	16 (30.18)	16 (30.76)	18 (34.61)	14 (26.92)	17 (32.69)
Occasionally	6 (11.32)	5 (9.61)	7 (13.46)	4 (7.69)	6 (11.53)
Sometimes	9 (16.98)	9 (17.30)	8 (15.38)	11 (21.15)	7 (13.46)
Most of the time	14 (26.41)	14 (26.92)	10 (19.23)	16 (30.76)	12 (23.07)
Do not know	8 (15.09)	8 (15.38)	9 (17.30)	7 (13.46)	10 (19.23)
Total	53 (100)	52 (100)	52 (100)	52 (100)	52 (100)

A notable proportion of participants who were afflicted with the aforementioned negative feelings reported some degree of improvement after the implementation of SafeHome modifications (see Table 7). The most substantial improvement was observed in the proportion of respondents who reported some degree of reduction of stress (over 80%) and anxiety (over 70%).

**Table 7:** Improvement on negative feelings after the implementation of SafeHome modifications, Count (%)

	Stress	Sleeplessness	Depression	Anxiety	Unhappiness
No change	7 (18.91)	11 (30.55)	13 (38.23)	10 (26.31)	14 (40)
A little	12 (32.43)	7 (19.44)	7 (20.58)	8 (21.05)	5 (14.28)
Some	5 (13.51)	6 (16.66)	5 (14.70)	6 (15.78)	7 (20)
Mostly	4 (10.81)	4 (11.11)	3 (8.82)	5 (13.15)	3 (8.57)
Definitely	9 (24.32)	8 (22.22)	6 (17.64)	9 (23.68)	6 (17.14)
Total	37 (100)	36 (100)	34 (100)	38 (100)	35 (100)

#### Outcome 2 results: COST–benefit analysis of SafeHome

**Fig. 2:**
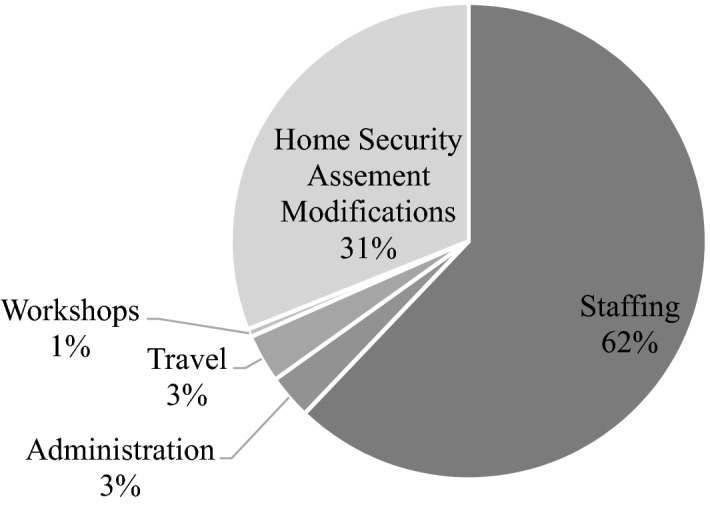
Percentage breakdown of costs

The costs associated with the SafeHome Program between the period July 2018 and June 2020 are provided in Table 8. The actual costs of the program reveal a deficit (i.e. costs spent in excess of grant received). Disaggregated, SafeHome Program costs include: staffing (AUD111,591.65), administration (AUD5418.42), travel (AUD5903.99), workshops (AUD985.98), and home security assessment modifications (AUD55,757.77) (see Fig. 2 for percentage breakdown of costs). On average, each client costs the SafeHome Program approximately AUD129.94 and AUD150.25 for the supply (e.g. a lock) and installation of security devices by a contractor, respectively (i.e. total modification costs divided by number of clients who requested modifications based on assessment).

**Table 8:** Actual costs of SafeHome Program between July 2018 and June 2020

	First report 1/07/18–30/06/19	Second report 01/07/19–30/06/20	Final report 01/07/18–30/06/20
Total income (GST exclusive)			
Grant	$95,000.00	$80,000.00	$175,000.00
Surplus funds carried over from the previous period	$0.00	$9214.18	
Total income	$95,000.00	$89,214.18	$175,000.00
Total expenses (GST exclusive)			
Staffing			
Home safety coordinator	$47,300.00	$48,253.06	$95,553.06
Administrator	$543.00	$498.00	$1041.00
Management	$713.00	$740.00	$1453.00
Salary on costs	$6615.84	$6928.75	$13,544.59
Subtotal	*$55,171.84*	*$56,419.81*	*$111,591.65*
Administration			
Rent	$1593.00	$1593.00	$3186.00
Finance/accounting, legal (contract review), audits	$416.00	$398.00	$814.00
Insurances	$257.00	$231.00	$488.00
IT/communications/office supplies	$284.00	$646.42	$930.42
Subtotal	*$2550.00*	*$2868.42*	*$5418.42*
Travel			
Vehicle operating expenses	$3128.00	$2775.99	$5903.99
Subtotal	*$3128.00*	*$2775.99*	*$5903.99*
Workshops (12practical community workshops)			
Printing, development of resource, resource manuals	$512.98	$473.00	$985.98
Materials (materials in stock already)	$0.00	$0.00	$0.00
Subtotal	*$512.98*	*$473.00*	*$985.98*
Home Security Assessment Modifications			
Modifications supplies (est $120 average per residence)	$13,880.00	$11,978.17	$25,858.17
Modifications Install—Contractor (est. 257 residence)	$10,543.00	$19,356.60	$29,899.60
Subtotal	*$24,423.00*	*$31,334.77*	*$55,757.77*
Total Expenses	$85,785.82	$93,871.99	$179,657.81
Surplus / Deficit (Total Income minus Total Expenses)	$9214.18	− $4657.81	− $4657.81

Italic relates to subtotals of figures to assist in their easy identification

In the first year of the program (1/07/18–30/06/19), there was a surplus of AUD9214.18 (grant income = AUD95,000, expenses = AUD85,785.82). In the second year of the program, there was a deficit of AUD4657.81 (grant income and surplus carried forward = AUD89,214.18, expenses = AUD93,871.99). According to service provider reports, the deficit can be attributed to: (i) an increase in office expenses due to COVID requirements at the facilitators’ office and on clients’ residence; (ii) a wage increase due to Fair Work Wage increases and additional travel time required; and (iii) the higher installation costs due to an increase in contractor supply costs and additional travel costs.

#### Participant willingness-to-pay for SafeHome

Survey responses to the willingness-to-pay (WTP) questions reveal that participants were willing to pay on average no less than AUD62.77 for the face-to-face home safety assessment and no less than AUD257.08 for the SafeHome changes and modifications made to enhance security (see Table 9). The lower bound has been adopted for the subsequent analysis to present a conservative estimate of the benefit-to-cost ratio for using SafeHome to enhance security.

**Table 9:** Willingness-to-pay estimates for SafeHome

	Count (%)	Aggregated lower bound WTP (AUD)	Aggregated upper bound WTP (AUD)
Willingness to pay to receive the face-to-face security assessment			
$0	14 (26.42)	0	0
Up to $50	12 (22.64)	12	600
$51 to $100	10 (18.87)	510	1,000
$101 to $200	6 (11.32)	606	1,200
$201 or more	11 (20.75)	2,211	2,211 or above
Total	53 (100)	3,327	5,011 or above
Average of individual WTP		62.77	94.55 or above
Willingness to pay to for the SafeHome changes and modifications made to enhance security			
$0	13 (24.53)	0	0
Up to $100	15 (28.30)	15	1,500
$101 to $500	11 (20.75)	1,111	5,500
$501 to $1000	3 (5.66)	1,503	3,000
$1001 or more	11 (20.75)	11,011	11,011 or above
Total	53 (100)	13,625	21,011 or above
Average of individual WTP		257.08	396.43 or above

Both the aggregated lower and upper bounds represent the total WTP. For example, 12 respondents were willing to pay ‘up to $50’. Therefore, the aggregated WTP ranges from $12 (lower bound) to $600 (upper bound—12*50)

The average represents the aggregate of all individual WTP divided by the number of respondents

With the assumption that survey participants are representative of the overall SafeHome participant population, we have applied the survey data to estimate the total WTP of the participant population. In sum, all SafeHome participants combined, during the reference period, would be willing to pay at least AUD21,279 for the face-to-face security assessment (i.e. AUD62.77*339 households which received a home security assessment) and at least AUD50,644 for enhanced security as a result of SafeHome (i.e. AUD257.08*197 households which made changes and modifications to enhance security).

The aggregate WTP per person (i.e. no less than AUD319.85 (AUD62.77 + AUD257.08)) outweighs the average costs per SafeHome participant (i.e. AUD280.19 (AUD129.94 + AUD150.25). Therefore, the benefit-to-cost ratio is *1.14* suggesting that every dollar spent on the program results in no less than AUD1.14 of benefits. It is our expectation that this is a very conservative estimate of the return on investment from the program as it does not include the monetisation of other enhanced aspects of quality of life or indirect impact on outcomes such as increased property prices and improvements in retail sales, etc. Since the results in outcome 1b suggest that SafeHome might have contributed to preventing one domestic burglary among survey participants, the estimated avoided costs of burglary can be added as a benefit of the program. Converting from the UK costs of crime data, an avoided domestic burglary generated an estimated saving of AUD2275.48 (GBP1270 (Heeks et al. 2018)) (1AUD = 0.56GBP – exchange date 29/01/2021) to society. The benefit-to-cost ratio which takes into consideration of the potentially avoided burglary is *1.29 *(i.e. AUD362.78/AUD280.19). Therefore, every dollar spent on the program produces no less than AUD1.29 in benefits.

#### Participant willingness to pay in hypothetical prevention scenarios

Two hypothetical scenarios were presented to participants. The aim was to elicit their WTP to reduce their risk of re-victimisation by a given amount. Participants’ WTP responses to these scenarios (i.e. SafeHome modifications reducing 50% and 90% of re-victimisation risk) reflected an overall logical and consistent WTP where participants had a greater WTP when the security measures were able to reduce more risk (see Table 10).

**Table 10:** Willingness to pay estimates in hypothetical scenarios

	Count (%)	Lower bound (AUD)	Upper bound (AUD)
Willingness to pay for SafeHome modifications to reduce 50% of victimisation risk			
$0	15 (28.30)	0	0
Up to $100	12 (22.64)	0	1200
$101 to $500	11 (20.75)	1111	5500
$501 to $1000	8 (15.09)	4008	8000
$1001 or more	7 (13.21)	7007	7007 or above
Total	53 (100)	12,126	21,707 or above
Average per household		228.79	409.57 or above
Willingness to pay for SafeHome modifications to reduce 90% of victimisation risk			
$0	13 (24.53)	0	0
Up to $100	13 (24.53)	0	1300
$101 to $500	10 (18.87)	1010	5000
$501 to $1000	8 (15.09)	4008	8000
$1001 or more	9 (16.98)	9009	9009 or above
Total	53 (100)	14,027	23,309 or above
Average per household		264.66	439.79 or above

## Discussion

Overall, it appears that the SafeHome program produces positive benefits overall in terms of enhanced security (Outcome 1a), reduced risk of re-victimisation (Outcome 1b), improvement in perceived sense of personal security (Outcome 1c) and positive economic return on investment (Outcome 2).

A comparison of security configurations adopted by burglary victims before and after SafeHome revealed that the involvement of SafeHome was critical in enhancing household security. As a result of the initial security assessment and subsequent modifications (Outcome 1a), most participants improved their household security from less than basic to basic or above. According to UK evidence on corresponding burglary victimisation risk against different security configurations, households with ‘basic’ and ‘enhanced’ security with locked window and doors are at least 12.5 times less likely to be burgled and 7.6 times less likely to be a victim of attempted burglary when compared to households with ‘no security’ (Tseloni et al. 2016). While this evidence may not be directly translated to the Australian context, it demonstrates that modifications that improve household security has the potential to reduce the risk of burglary victimisation. Such potential is revealed by Outcome 1b where 53 households within the 38 suburbs was re-victimised, reflecting a re-victimisation rate of approximately 1.9% – which is lower than that of all 38 suburbs overall rate (3.1%).

Many participants also reported not being in a position to afford security improvements without the support of SafeHome. The survey also revealed that respondents felt safer when walking in their neighbourhood and staying at home. Participants also attributed different degrees of improvements in their psychological state to the SafeHome modification. Such a positive impact is shown in results (Outcome 1c), highlighting a decrease in the number of participants who were concerned about future break-ins and an increase in the number of participants who felt safe living and residing in their community (especially when they are at home during the day and at night). Program participants were asked how the program changed their perceptions and feelings, however, it should be noted that these perceptions and feelings may not be fully attributed to the SafeHome program alone and recognise that future victimisation would affect people’s perceptions. We encourage future research to fully examine this issue by investigating the longitudinal impact of target hardening and revictimisation on the perceptions and feelings over time. Such research is important as Manning and Fleming (2017) found that individuals’ perceptions of crime in their local area are far greater than actual levels of crime, where real crime rates detract more from an individual’s self-reported life satisfaction than perceived rates of crime. However, perceived rates of crime have an adverse impact on life satisfaction beyond those associated with real crime. Therefore, societal welfare could be enhanced by reducing individual’s perceptions of crime, which SafeHome appears to provide.

Regarding the lower re-victimisation rate as presented in Outcome 1b, SafeHome participants could be regarded as a particularly vulnerable group to re-victimisation compared to other victims within the suburb in which they reside. This may be due to their financial ability to implement changes to their level of security within a short period of time. This circumstance is revealed in Outcome 1a. Hence, the benefit of having a program such as SafeHome improves the chances of disadvantage groups (e.g. financially restrained) within a suburb or area of not being re-victimised. Although an at-risk group within a suburb may only represent a small proportion of those who reside in a suburb, benefits (both direct and indirect) may be extended to the greater community through an overall enhanced sense of security and morale (Laycock and Tilley 1995), positive impact to property prices (Ihlanfeldt and Mayock 2010) and wellbeing (Cornaglia et al. 2014).

Results of Outcome 2 reveal that the SafeHome program generated benefits which outweighed its costs. Benefits were captured by participants’ WTP for the face-to-face security assessment and subsequent modifications which they received from SafeHome. The method monetised participants’ perceived improvement in the overall sense of security as a result of SafeHome assessment and modifications. The resulting benefit-to-cost ratio of no less than 1.14 (or 1.29 when including potential avoided burglary) reveals that there are positive economic benefits associated with the program. A comparison of participant actual WTP and WTP for the two hypothetical scenarios suggests that, at the time of data collection, SafeHome participants perceived a benefit of enhanced security higher than that of the value of a 50% reduction in risk of victimisation. We note that a longer follow-up timeframe as well as the incorporation of other indirect benefits would most likely result in a larger benefit-to-cost ratio. We expect, similar to the evaluation performed by Bowers et al. (2004), that economic benefits derived from burglary prevention interventions tend to increase overtime as the intervention has time to mature.

### Final thoughts

We note that there are a number of limitations associated with this evaluation, with the most obvious being a dependent data collection process, a non-experimental research design and missing data. Although the data analyses were conducted independently by the Australian National. University Centre for Social Research and Methods, data for this evaluation were collected by the service provider (i.e. SupportLink). Some may argue that the data may be affected due to social desirability bias. Due to time restraints we were not in the position to control for this potential bias. We propose that future evaluations consider this issue.

Second, with regard to the measure of re-victimisation risk, future research should consider employing a quasi-experimental design using police crime data at the household level. With the use of local crime data, information on whether households in the population (i.e. program participants and eligible comparison group households) experienced repeat victimisation within a given time period could be ascertained. In this study we were unable to access these data and adopted the best possible alternative given data limitations.

Third, there was a subgroup of the SafeHome participant population that were not fully captured in the survey. These were clients who received an assessment under the SafeHome program but whose income was above AUD40,000 (i.e. 101 out of 339 clients). To our knowledge, we note that only 2% of this subgroup made modifications after the assessment. Such a low percentage may be attributed to two factors: (i) clients may have decided to make modifications outside of the program as no subsidies were provided to this group; and (ii) clients may not have seen immediate benefits from spending money to make modifications that arguably would reduce their risk of re-victimisation. More data are required to follow-up this subgroup to ascertain their decision to enhance security and their WTP for changes that may lower the probability of being re-victimised.

Fourth, the EMMIE framework (Johnson et al. 2015) identifies five dimensions which individual evaluations of crime prevention initiatives should consider. These dimensions include the *E*ffect of intervention, the causal *M*echanism(s), the factors that *M*oderate intervention impact, the articulation of practical *I*mplementation issues, and the *E*conomic costs of the intervention. We encourage future evaluations to aspire to reaching the highest standard with regard to the EMMIE framework across all dimensions. This would require careful consideration of all dimensions in the intervention and evaluation design prior to intervention inception.

Finally, future evaluations of the SafeHome program should consider capturing the benefits of the SafeHome Workshops. Again, this is expected to contribute to the economic and psychological benefits associated with the program. This will allow for the holistic evaluation of the overall societal costs and benefits of the program.

## Footnotes

1 Property crime decreased across all states and territories during 2020, with a decrease of victimisation when COVID-19 measures first came into effect.

2 Psychological conditions include stress, sleeplessness, depression, anxiety and unhappiness.

3 The reason for requesting the service provider to administer the survey is to protect the identity of participants in the program. These anonymous data were managed independently by the project team at the ANU. All data are secured in a password protected USB device that is secured in a locked cabinet in a locked office.

4 As proposed by Kleemans (2001).

5 Here, d refers to Cohen’s d. Cohen’s d is a common way to measure an effect size. An effect size is how large an effect of something is. For example, intervention A has a better effect than intervention B. As a general rule of thumb, a d of 0.2 represents a small effect, 0.5 represents a medium effect, and 0.8 represents a large effect (Rosenthal, Cooper, and Hedges 1994).
